# Breaking Barriers: Modulation of Tumor Microenvironment to Enhance Bacillus Calmette–Guérin Immunotherapy of Bladder Cancer

**DOI:** 10.3390/cells13080699

**Published:** 2024-04-18

**Authors:** Omar M. Ibrahim, Pawel Kalinski

**Affiliations:** 1 Department of Medicine, Washington University School of Medicine, St. Louis, MO 63110, USA; oibrahim@buffalo.edu; 2 Department of Immunology, Roswell Park Comprehensive Cancer Center, Buffalo, NY 14263, USA

**Keywords:** tumor microenvironment, BCG, bladder cancer, urothelial cancer, chemokines, cytokines, immunotherapy, inflammation, lymphocytes, macrophages, Toll-like-receptors

## Abstract

The clinical management of bladder cancer continues to present significant challenges. Bacillus Calmette–Guérin (BCG) immunotherapy remains the gold standard of treatment for non-muscle invasive bladder cancer (NMIBC), but many patients develop recurrence and progression to muscle-invasive disease (MIBC), which is resistant to BCG. This review focuses on the immune mechanisms mobilized by BCG in bladder cancer tumor microenvironments (TME), mechanisms of BCG resistance, the dual role of the BCG-triggered NFkB/TNFα/PGE2 axis in the regulation of anti-tumor and tumor-promoting aspects of inflammation, and emerging strategies to modulate their balance. A better understanding of BCG resistance will help develop new treatments and predictive biomarkers, paving the way for improved clinical outcomes in bladder cancer patients.

## 1. Introduction

PD1/PD-L1 blockers have recently become a standard of care for patients with advanced bladder (urothelial) cancer [1,2,3,4,5], but the early stages of this disease have been treated with local (intravesical) Bacillus Calmette–Guérin (BCG) immunotherapy since 1976 as an adjuvant treatment of non-muscle-invasive bladder cancer (NMIBC) [6]. BCG is a key component of standard care for high-risk NMIBC patients following transurethral resection of the bladder tumor and an option for intermediate-risk NMIBC patients [7,8]. The guidelines from the European Association of Urology and the American Association of Urology suggest a risk-based approach to maintenance therapy duration. According to these guidelines, individuals with intermediate-to-high-risk disease should undergo maintenance therapy for at least one year and up to three years [8,9]. Intravesical BCG therapy has shown superior efficacy in reducing tumor recurrence and progression compared to no treatment or adjuvant intravesical chemotherapy [10,11]. However, over 40% of high-risk NMIBC patients do not respond to BCG treatment, and many more patients develop recurrence and progression to BCG-resistant disease [12,13]. The mechanisms underlying the failure of the anti-tumor effects of BCG therapy are not fully understood [14,15,16]. This review provides an overview of the currently acknowledged immune barriers to BCG efficacy and identifies the areas of need for future research.

## 2. BCG and Tumor Immunity

BCG infiltrates the urothelium and is taken up by macrophages, initiating local immune activation [17,18,19,20]. The dominant pathway for the early activation of macrophages involves pro-inflammatory cytokines. The resulting epigenetic modifications at regulatory sites that control inflammatory and metabolic genes lead to increased intracellular glycolytic metabolism and oxygen consumption in host macrophages [21,22,23]. These early events orchestrate gene transcription, resulting in an intensified cytokine production environment within the BCG-activated cells. This augmented cytokine response surpasses the initial wave, leading to increased recruitment and activation of cytotoxic leukocytes, ready to execute their defensive mission [24,25,26].

Innate immunity plays a crucial role in initiating the immune response against BCG therapy. In addition to BCG-activated macrophages, polymorphonuclear cells (PMNs), dendritic cells (DCs), and natural killer (NK) cells are involved in the response to BCG [27,28,29]. DCs, responsible for the induction of antigen-specific immunity, also activate innate immunity, promoting the killing of cancer cells by NKT cells and γ/δT cells [30,31]. In the context of BCG treatment of NMIBC, the effectiveness of BCG-induced immune responses and its potential anti-tumor benefits rely on the presence of subsequent immune challenges. These challenges play a crucial role in BCG-activated cells, leading to elevated production of Th1 cytokines and resulting inflammation [32,33,34].

In addition to innate immunity, adaptive immunity, particularly T cells, is critical to the effectiveness of BCG therapy [35,36,37]. In animal models and human studies, T cells, including CD4^+^ and CD8^+^ T cells, are found in the urine and bladder mucosa of BCG-treated patients [35,36,37]. BCG treatment was shown to induce a shift from a T helper 2 (Th2) to a T helper 1 (Th1) response, characterized by elevated levels of IL-2, IL-12, and interferon-gamma (IFNγ) [38,39,40]. This shift toward a Th1 response is associated with a favorable clinical response to BCG [40]. The role of T cells in BCG efficacy is demonstrated by the loss of BCG’s effectiveness in the absence of T cells in animal models [35,36]. BCG-induced T cell responses target both BCG antigens and bladder cancer antigens [41]. The transfer of T cells from BCG-cured mice to tumor-bearing mice results in tumor rejection, indicating the presence of tumor-specific memory T cells [35]. Preliminary human data also suggests the presence of tumor-specific T cells in BCG-treated patients [42].

The crucial role of the adaptive immune system in the susceptibility and resistance of bladder cancer to BCG therapy is demonstrated by the differential regulation of MHC class I on bladder cancer cells [16]. The initial response of cancer cells to BCG treatment typically includes an increased expression of MHC class I, which helps activate the infiltrating CD3^+^CD8^+^ T cells. However, a portion of cancer cells adapt by reducing the levels of MHC class I after BCG therapy, limiting the increases in T cells within the TME, accompanied by an enhanced immunosuppressive myeloid profile. The downregulation of HLA-I expression in bladder cancers is unlikely to result from immunoediting (immune-mediated selection of tumor cells with reduced immunogenicity), but it reflects a direct BCG-induced HLA-I loss in cancer cells [43,44,45,46,47,48,49], a part of a wide spectrum of suppressive events involving prostaglandins and elevation of PDL1 expression in bladder cancer TME [50,51,52], indicating the need for coordinated targeting of these pathways.

Limited research has addressed the role of B cells and humoral immunity in the response to BCG. Baseline levels of CD79a^+^ B cells before BCG treatment predict poor treatment outcomes in male and female NMIBC patients [53], suggesting that the expansion of exhausted immune cell populations, including atypical B cells, may contribute to early disease recurrence [53]. Paradoxically, while the presence of tertiary lymphoid structures (TLS) is typically associated with improved cancer outcomes [54,55], a recent study indicated that pre-BCG TLS presence is a negative prognostic factor in NMIBC [56]. Moreover, B cell depletion increased the production of IFNγ/IL-2 and IL-13 and the intratumoral levels of CD8A, CXCL10, CXCL9, and BATF3 in response to BCG treatment [56], which is consistent with CTL recruitment and a shift toward the desirable Th1/CTL-dominated immune landscape [56]. BCG treatment was associated with an overall elevation of TNF, IL-6, CXCR4, and CXCR5, the genes associated with the expansion of atypical B cells and TLS formation, suggesting their contribution to poor treatment outcomes [56], further suggesting that B cell depletion may have the potential to enhance the effectiveness of BCG treatment [56]. These findings underscore the emerging role of B cells in shaping the immune landscape, the treatment efficacy of BCG, and the need for the identification of the underlying mechanisms.

The clinical efficacy of BCG therapy is also affected by the molecular patterns of early TME responses to BCG. A recent study in high-risk NMIBC identified three molecular BCG response subtypes (BRS) predicting different clinical outcomes [57]. BRS1 represents TME that is favorable for BCG treatment due to the presence of the desirable effector immune cells. BRS2 predicts a moderate response to BCG, reflecting a balance between pro-tumorigenic and anti-tumorigenic immune cell populations within the TME. BRS3 reflects high activity of CD8^+^ T cells with indications of T cell exhaustion, leading to a poor clinical response to BCG immunotherapy [57]. These distinctions indicate a potential for using immune molecular subtypes in tailoring treatment approaches for bladder cancer patients undergoing BCG therapy by either modulating the TME to reduce its immunosuppressive components or selecting patients who are likely to respond to BCG treatment alone and those who need combination therapies.

## 3. BCG, Immune Checkpoints, and Immune Checkpoint Inhibitors (ICI)

The crucial immunological checkpoint maintaining immune homeostasis and moderating prolonged T cell responses is the interaction between the Programmed Death-1 (PD-1) molecule, expressed on T cells, and its ligands (PD-L1 and, to a lesser extent, PD-L2). PD-L1 is typically expressed by cancer cells and myeloid cells, such as macrophages, but it can also be induced on activated T, B, and NK cells, endothelial cells, and other non-malignant cells in an inflammatory environment. The overexpression of PD-L1 in cancer cells and surrounding stromal cells allows malignant cells to circumvent the immune response, leading to T cell inactivation [58,59]. The examination of tissue microarrays from pre- and post-Bacillus Calmette–Guérin (BCG) bladder samples revealed that 25–30% of patients who did not respond to BCG treatment exhibited intratumoral overrepresentation of PD-L1 at baseline, associated with high levels of CD8^+^ T cells, but low levels of CD4^+^ T cells [60,61]. In contrast, PD-L1 expression was virtually non-existent in patients who responded to BCG treatment [60,61]. These findings were corroborated by additional studies showing that PD-L1 expression in both tumor cells and immune cells was more pronounced in patients with BCG-unresponsive carcinomas in situ (CIS) compared to BCG-responders, suggesting that baseline PD-L1 expression could serve as a predictive marker for CIS that would not respond to BCG therapy [62,63]. Moreover, BCG treatment was found to augment the expression of both PD-L1 and PD-1. In a cohort of NMIBC patients treated with BCG, PD-1 expression was higher in BCG-unresponsive tumors compared to pretreatment tumors from the same patients, leading to the hypothesis that BCG could stimulate this immune checkpoint. BCG instillation appears to stimulate the expression of PD-L1 in tumor and inflammatory cells through the induction of CD8^+^ T cells, which are primarily responsible for IFN-γ production [64]. An increase in the number of PD-L1-expressing CD4^+^ T cells (PD-L1^+^ Tregs) was reported in BCG-resistant patients [65]. It was also demonstrated that BCG treatment triggers the up-regulation of PD-L1 expression on antigen-presenting cells (APCs), leading to the secretion of certain cytokines such as IL-6 and IL-10, which in turn lead to STAT3 phosphorylation and ultimately PD-L1 expression [66]. This aligns with a recent study that found that NMIBC patients exhibiting resistance to BCG therapy showed heightened levels of PD-L1^+^ cellular expression, which is in stark contrast to the negligible presence of these cells in patients who responded to BCG [67]. In the therapeutic context, PD-L1 positivity is advantageous when treatments are designed to target the PD-1–PD-L1 axis, as the lack of such targeted therapy can lead to tumor immune evasion. Consequently, both PD-1 and PD-L1 have potential as biomarkers to predict the response to BCG therapy. These observations provided rationale for clinical trials, evaluating the effectiveness of PD-1/PD-L1 blockade, either as the next stage of treatment for patients who progressed on BCG or in combination with BCG in high-risk NMIBC (Table 1).

In the recently completed randomized clinical trial, KEYNOTE-057, Pembrolizumab (Keytruda; PD-1 blocker) has shown positive results in high-risk NMIBC unresponsive to BCG, culminating in its recent approval by the FDA [76,77,78]. The trial reported a favorable safety profile [78] and 41% a complete response (CR) rate at the 3-month mark among the 96 patients with BCG-unresponsive carcinoma in situ (CIS) with or without papillary tumors (Cohort A). Nivolumab, a PD-1 inhibitor previously approved for other indications, has recently received FDA approval as a part of second-line therapy for metastatic bladder cancer (BC), setting the stage for its exploration as a new treatment for BCG-resistant high-risk non-muscle-invasive bladder cancer (HR-NMIBC) [79,80].

A phase 3 randomized study, CheckMate 7G8 (NCT04149574), is currently testing the efficacy of Nivolumab in conjunction with BCG, in comparison to BCG monotherapy, in patients who have persistent or recurrent high-risk NMIBC following a single adequate course of BCG induction. The primary endpoint of this trial is event-free survival (EFS) [68]. Sasanlimab, another inhibitor of the PD-1/PD-L1 interaction that binds directly to PD-1 [69], is currently being evaluated in a phase III trial (NCT04165317) to assess its efficacy in combination with alternative BCG regimens in high-risk non-muscle-invasive bladder cancer (HR-NMIBC) [69]. The trial is divided into two cohorts. Cohort A includes BCG-naïve participants and is further divided into three arms (A, B, and C). Arms A and B, which involve the administration of BCG in conjunction with Sasanlimab, are evaluated against arm C, which employs BCG monotherapy for both induction and maintenance phases. Cohort B is composed of patients unresponsive to BCG with carcinoma in situ (B1) or papillary disease (B2) [69]. The primary endpoints of the study are event-free survival (EFS) and complete response (CR) rates. The trial’s completion is expected in 2026.

Durvalumab is a monoclonal antibody targeting PD-L1, thus blocking the PD-1/PD-L1 interaction from the other end, without directly engaging PD1 but directly interacting with cancer cells and local myeloid cells [81]. In a phase I clinical study (NCT03317158), durvalumab was administered with either BCG or external beam radiation therapy (EBRT) in NMIBC patients with BCG-unresponsive, BCG-relapsing, and high-risk BCG-naive (HR-NMIBC) disease, demonstrating a 12-month complete response (CR) in 46% of the total patient cohort, with a notable 73% CR in the durvalumab plus BCG subgroup and a 33% CR in the durvalumab plus EBRT subgroup [70]. The study’s primary focus was on establishing the recommended phase 2 dose (RP2D). Currently, a phase III trial (NCT03528694) is examining the efficacy of durvalumab in combination with BCG in BCG-naive patients [71]. Another phase II trial (NCT03759496) is assessing the safety and effectiveness of durvalumab in patients with BCG-refractory NMIBC, with the maximum tolerated dose (MTD) and 1-year high-grade relapse-free rate as primary outcomes [72]. Further evaluation of durvalumab is taking place in the phase I/II RIDEAU study (NCT05120622), which aims to determine the efficacy of systemic durvalumab in conjunction with the anti-CTLA-4 antibody (Tremelimumab; blocker of the inhibitory interaction between CTLA4 and B7.1/B7.2) in HR-NMIBC patients [73]. Lastly, the phase I/II DURANCE trial (NCT04106115) tests the combination of durvalumab with S-488210/S-488211, a five-peptide vaccine designed to elicit a cytotoxic T-lymphocyte response and promote tumor cell lysis [74].

Atezolizumab, a monoclonal anti-PD-L1 antibody blocking the PD-L1/PD-1 pathway, has been evaluated in 24 patients with BCG-unresponsive non-muscle-invasive bladder cancer (NMIBC) in a non-randomized phase Ib/II clinical trial (NCT02792192) [75,82,83]. The results indicated that 33.3% of patients treated with Atezolizumab alone and 41.7% of patients treated with a combination of Atezolizumab and BCG achieved complete remission (CR) at the 6-month follow-up mark [75,83]. In the most recent phase III study, ALBAN, 516 patients were randomized across 45 centers in Europe at a 1:1 ratio between arm A (BCG control) and arm B (BCG plus atezolizumab) [84].

## 4. BCG and Lymphoid/Myeloid Imbalance

The local balance between T lymphocytes and myeloid suppressor cells (MDSCs) is critical for the outcomes of BCG therapy, independent of patient status, disease stage, and histologic types of the tumor [85]. Of additional importance is the effector-to-regulatory T cell balance, highlighting the role of FoxP3^+^ regulatory T cells (Tregs), which show prognostic value independently from MDSCs [86,87]. These observations indicate that the levels of effector and memory T cells in relation to Tregs and MDSCs in urine may serve as a predictive biomarker of the therapeutic efficacy of BCG.

While MDSCs have been extensively studied in mouse cancer models, their clinical prognostic value in human cancers, including bladder cancer, is less well established [88,89]. High infiltration of CD68^+^ tumor-associated macrophages (TAMs) has been associated with poor response to BCG immunotherapy (37). Interestingly, only their levels in the tumor itself but not in the adjacent lamina propria predict the failure of BCG therapy for bladder carcinoma in situ (CIS) [89,90,91].

Recent studies in murine tumor models have indicated an important role of innate lymphoid cells (ILCs) in the outcomes of anticancer immunity [92,93]. ILC1s and potentially ILC3s have been implicated in tumor immunosurveillance, while ILC2s have been shown to be detrimental [94]. Intriguingly, the presence of ILC2s, both locally in the TME and in the peripheral blood of bladder cancer patients, strongly correlated with disease progression [85]. A positive correlation between ILC2 and monocytic (M)-MDSC levels was identified, both locally during BCG therapy and in the blood of patients with muscle-invasive bladder cancer (MIBC) [85]. The molecular link between these cell subsets appears to be IL-13, as M-MDSCs recruited to the bladder highly express IL-13Rα1, and ILC2s secrete IL-13 in response to BCG and tumor cells in vitro. Furthermore, urine samples with detectable IL-13 exhibited higher frequencies of ILC2s, suggesting a potential role for IL-13 in ILC2s recruitment. Additionally, IL-13 was found to preferentially recruit and induce suppressive function in monocytic cells, possibly mediated by ARG1, an enzyme highly expressed in urine M-MDSCs [85].

Therefore, in addition to promoting a robust Th1 response, which may depend on preexisting BCG-specific adaptive immunity, BCG therapy may amplify an existing immunosuppressive TME involving MDSCs and ILC2s [41,95,96,97,98], indicating the importance of restraining the tumor-induced “immunosuppressive switch” by targeting these cells to shift the balance toward Th1/CTL responses.

## 5. BCG and Chemokines Attracting Effector versus Suppressive Populations of Immune Cells: Rationale for Modulating PGE2 Production and Signaling

Suppressive TME has been shown to be an important factor contributing to ICI unresponsiveness [99,100]. In addition to immune checkpoints, two other areas of immunosuppressive activity of the TME have been postulated to be key to its reduced ability to support anti-tumor activity of immune cells: (a) reduced attraction of type-1 immune cells (CTLs, Th1, and NK cells) associated with enhanced influx of immunosuppressive cells, such as Tregs and MDSCs; and (b) enhanced production of suppressive factors by TME-resident and newly infiltrating myeloid and stromal cells. Data from us and other labs demonstrate the key role of an arachidonic acid metabolite and cyclooxygenase (COX) product, prostaglandin E2 (PGE2) [101,102,103,104,105], in the orchestration of both these negative aspects of the suppressive TME.

Tumor-derived PGE2 has been shown to be responsible for local dysfunctional DCs within the bladder TME, undermining their ability to support infiltrating CD8^+^ T cells and resulting in ineffective immunity and immune escape [50]. Preventing such PGE2-induced dysfunction in DCs restores effective T cell-mediated control of tumor growth. PGE2, produced by cyclooxygenases, acts through cAMP-inducing EP2 and EP4 receptors in DCs [50,101] and other myeloid cells, including a shift from M1 to M2 macrophages and induction, attraction, and activation of MDSCs [51,52,101,102,103,104,106,107].

Our data from bladder and other cancer types (colorectal, prostate, and ovarian cancers) [51,108,109,110] demonstrated that high COX2 levels are associated with the suppression of CTL-attracting chemokines and the overexpression of Treg attractants and that its suppression can promote selectively enhanced attraction of type-1 immune cells while inhibiting Treg and MDSC attraction [103]. Interestingly, untreated bladder cancer TMEs showed nearly a complete lack of effector cell-attracting chemokines but selective expression of Treg/MDSC attractants IL-8/CXCL8, CXCL-12, and CCL22 [51,111].

**Figure 1:**
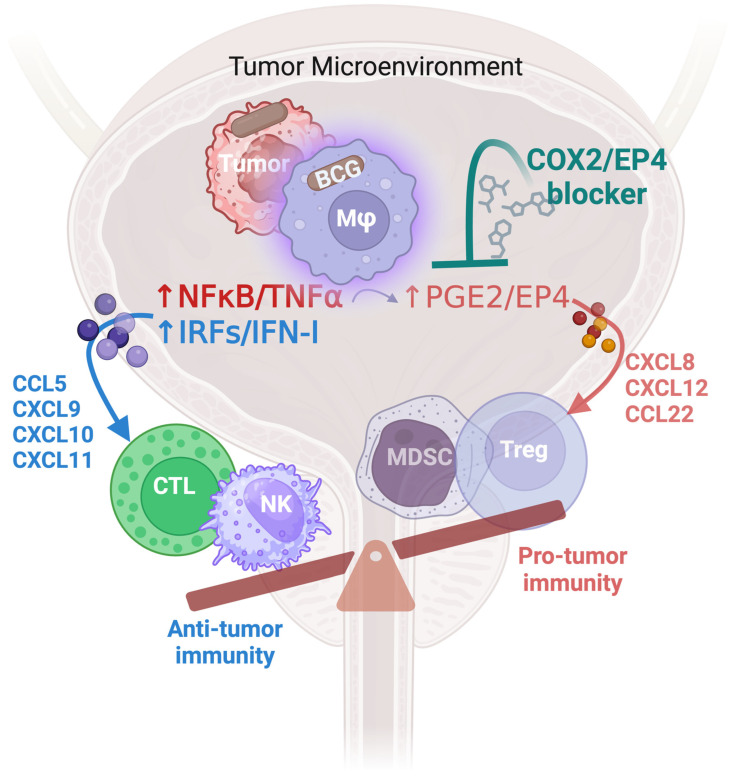
BCG-driven activation of the bladder cancer TME involves the NFκB/TNFα signaling pathway and IRFs/IFN pathway. While the IRF/IFN pathway selectively induces the chemokines attracting the desirable effector cells, the NFκB/TNFα pathway enhances the induction of both the CTL-attracting cytokines, CCL5, CXCL9, CXCL10, and CXCL11, but also amplifies the undesirable COX2/PGE2/EP4 pathway, which orchestrates the induction, activation, and recruitment of MDSCs and Tregs, through chemokines such as CXCL8, CXCL12, and CCL22. The combination of BCG with COX2- or EP4 blockers can selectively augment the attraction of CTLs while neutralizing PGE2-dependent suppressive factors and Treg and MDSC attractants, suggesting its potential to augment effective anti-tumor immunity in response to BCG treatment.

We observed that BCG-induced inflammation in human bladder cancer tissues involves the induction of COX2 and its product PGE2, associated with the EP4-mediated induction of the chemokines CCL22 and CXCL8, which attract myeloid-derived suppressor cells (MDSCs) and regulatory T cells (Tregs) [51,111]. Blockade of PGE2 synthesis or EP4-mediated signaling eliminated these undesirable effects, instead enhancing the BCG-driven induction of CTL-attracting chemokines, such as CCL5, CXCL9, and CXCL10 (Figure 1), associated with the differential impact of attraction of CTLs versus Tregs in preclinical models [51]. Interestingly, BCG treatment was also associated with elevation of additional COX2/PGE2-dependent suppressive factors, such as indoleamine-2,3-dioxygenase 1 (IDO1) and IL10 [51,111,112], which is consistent with our earlier observations that COX2 is critical for the induction, persistence, and suppressive activity of tumor-associated MDSCs [102,104]. These observations raise the possibility that manipulating the chemokine system, or, more broadly, prostaglandin antagonism, may be used to enhance the efficacy of BCG therapies and counteract BCG unresponsiveness.

BCG-triggered immune responses are affected by the levels of indoleamine 2,3-dioxygenase 1 (IDO1) in the TME [113,114,115]. IDO1-mediated tryptophan catabolism results in local tryptophan depletion, impeding T cell function and proliferation, thereby attenuating the BCG-induced immunity [113,114,115]. Moreover, IDO-produced metabolites of the kynurenine pathway suppress effector T cell function and promote the development of regulatory T cells (Tregs), thereby fostering an immunosuppressive TME [115]. Kynurenine, an IDO product, activates the aryl hydrocarbon receptor (AhR), facilitating the differentiation of T cells into Tregs and contributing to immune tolerance [115]. The genetic makeup of individuals regulates IDO1 expression through the polymorphisms or mutations that regulate the IDO1 gene directly or modulate the regulatory pathways governing IDO1 expression [116,117,118]. Polymorphisms associated with heightened IDO1 activity, fostering a suppressive TME that is conductive of immune evasion [116,117,118], indicating their consideration in the therapeutic potential of IDO1-targeted interventions [119,120]. Furthermore, chemotherapeutic agents can elevate IDO1 expression as a result of the cancer cell stress response [121,122]. Conversely, other therapeutic modalities may mitigate IDO1 expression or its immunosuppressive effects, thereby influencing subsequent responses to BCG [121,122]. Combining BCG therapy with IDO1 inhibitors or other immunotherapeutic modalities, including ICI, holds promise for augmenting the overall outcomes in NMIBC.

IFNα, which suppresses EP4 expression and antagonizes PGE2-driven immune suppression [106], has demonstrated the ability to promote anti-tumor immunity in various bladder cancer models [123,124]. However, the combination of BCG and IFNα did not exhibit any advantage over BCG monotherapy in patients with relapsed non-muscle-invasive bladder cancer (NMIBC) [125,126]. These disappointing results may be attributed to the observation that the combination of IFNα with BCG, alone or with poly-IC (which activates not only the IFN-enhancing Toll-like receptor 3 pathway but also the COX2-augmenting RIG-I/MDA5/NFkB pathway), not only enhances CXCL10 production but also triggers the production of CCL22 in human tumor tissues [106,111].

Notably, the local inflammatory response triggered by BCG administration in bladder cancer patients is reflected by the increased presence of macrophages, T cells, B cells, natural killer (NK) cells, and neutrophils in the urine [127]. These elevated cell levels and the levels of PD1^+^ T cells have been shown to predict the clinical response to ICI combined with BCG [67], which is well aligned with the observations that the overall levels of CD8^+^ T cells in the TME correlate with improved survival in bladder cancer patients [128]. Conversely, elevated levels of Tregs in the TME and urine in response to BCG predict poor treatment outcomes [129,130]. Similar, the predominance of MDSCs over T cells following BCG therapy predicts reduced recurrence-free survival [85].

These observations help explain the ability of COX1 and COX2 inhibitors to overcome resistance to BCG in mouse models [52,131,132,133], providing rationale for in-depth evaluations of PGE2 interference as a potential tool to improve the efficacy of BCG (Figure 1).

It is currently unclear how far the COX2/PGE2 system is involved in the induction and maintenance of PD-L1 and PD-L2 expression on cancer cells and cancer-associated myeloid and stromal cells, but these two inhibitory pathways appear to be at least partially independent, resulting in a synergistic effectiveness of their blockade in preclinical mouse models [134,135,136,137,138]. Synergistic activities were also reported between immune checkpoint inhibition and inhibitors of IDO, a downstream mediator of PGE2-orchestrated immune suppression. Dual blockade of CTLA-4 or PD-1/PD-L1 combined with IDO inhibition proved to be highly effective in enhancing the TME-infiltration with CD8^+^ T cells [139]; however, a phase II l trial, Check-Mate 9UT (NCT03519256), designed to assess the anti-tumor efficacy of an oral IDO inhibitor, BMS-986205, in combination with nivolumab in patients with high-risk BCG-unresponsive NMIBC, was discontinued due to poor enrollment [140]. Therefore, the potential for enhancing the effectiveness of both ICI and BCG therapies (as well as their combinations) using PGE-blocking strategies remains to be investigated.

## 6. Beyond Cyclooxygenase Inhibitors: Emerging Targets and Biomarkers to Counteract PGE2-Driven Suppression and Enhance Type-1 Inflammatory Pathways

BCG activates two main inflammatory pathways: the TRIF/IRF3/IFNα/β pathway, which has a uniformly pro-immunogenic and anti-tumor role, and the NFκB/TNFα signaling pathway, which mobilizes immunogenic/anti-tumor but also immunosuppressive/tumor-promoting mediators in bladder cancer TME. Since BCG and BCG-expressed Toll-like receptors 2, 4, 9, and Mincle all signal through the myeloid differentiation primary response 88 (MyD88) and NFκB pathway, which is needed for the induction of TNFα and PGE2 and not only promotes immune suppression but also metastasis and resistance to apoptosis [51,112,141,142,143,144,145,146], using the existing blockers of NFκB- and TNFα signaling represents additional tools that may enhance the effectiveness of BCG therapy and its combinations.

The downstream immunomodulatory effects of PGE2 are mediated through its receptors EP1, EP2, EP3, and EP4 [101]. Among these, EP2 and EP4, which both activate adenylate cyclase, leading to cAMP elevation and activation of protein kinase A (PKA) and CREB phosphorylation, have been identified as key mediators of immune suppression and tumor promotion in multiple cancer types [101]. Notably, the overexpression of the EP4 receptor, observed following BCG treatment, and cyclooxygenase-2 (COX2) has been linked to poor overall survival in bladder cancer patients [147]. PGE2 drives an EP4-mediated upregulation of COX2, establishing a positive feedback loop involving COX2, PGE2, and EP4. This loop is critical for driving excessive intratumoral production of not only PGE2 but also multiple PGE2-dependent “secondary” suppressive factors, including IDO1, IL-10, and ARG1, which inhibit the activation, expansion, attraction, and effector functions of cytotoxic T lymphocytes (CTLs) and natural killer cells (NKs) (Figure 1). Therefore, targeting each of these elements of PGE2 signaling offers an opportunity to counteract multiple elements of TME-associated suppressive phenomena, both at baseline and in the course of immunotherapy, offering an advantage over targeting individual suppressive factors.

Significantly, elevated production of intratumoral PGE2 translates into increases in PGE2 levels within the urinary tract, which are augmented after BCG immunotherapy [43,47]. Therefore, measurements of PGE2 levels (or its metabolites) in urine could serve as indicators of local PGE2-mediated suppression and be used as a surrogate measurement of the effectiveness of PGE2-targeting strategies used in combination with BCG and/or immune checkpoint inhibitors.

## 7. Conclusions and Perspectives

The intricate interplay between bladder cancer TME and BCG and other forms of cancer immunotherapy involves both immuno-activating and immunosuppressive elements of immunity, jointly affecting the response to treatment of individual patients and affecting its diminishing effectiveness in the course of bladder cancer progression. While BCG alone is the most commonly used approach, its efficacy is currently limited to a group of patients with NMIBC. The strong association between the levels of COX2 in BCG-activated myeloid cells and the local production of factors that attract regulatory T cells (Tregs) and myeloid-derived suppressor cells (MDSCs), as well as suppressive factors, indicates multiple opportunities for targeting the individual elements of the PGE2 induction and production pathway and its downstream signaling pathways to reprogram bladder cancer TME for enhanced effectiveness of BCG, ICI, and other forms of immunotherapy of NMIBC and potentially MIBC.

Notably, BCG induces a dual response, involving the enhancement of both desirable and undesirable immune mechanisms. Undesirable effects include the elevation of MDSC- and Treg-attracting chemokines, as well as multiple myeloid cell-produced suppressive factors, as a consequence of the activation of NFκB, TNFα, COX2, and suppressive PGE2 receptors. We propose a strategy to selectively modulate this response. Employing inhibitors of key molecules involved in PGE2 induction, synthesis, and signaling has the potential to suppress the induction of MDSC/Treg attractants and immunosuppressive factors produced by these cells while enhancing the production of chemokines that attract CTLs, Th1, and NK cells and facilitating their anti-tumor effector functions.

Since the balance between CTLs and suppressive cells within the TME is a strong prognostic factor for bladder cancer outcomes, correcting it is likely to correct the limitations of BCG therapies, prolonging their duration and possibly extending their efficacy to MIBC when used alone or as a part of combinatorial treatments involving other forms of immunotherapy, such as ICI, or possibly with chemotherapy, also known to depend on intratumoral CTLs, both in bladder cancer and possibly potentially additional malignancies. In accordance with this possibility, our recent clinical trials involving systemic blockade of COX2 (oral celecoxib), combined with the local- or systemic administration of the chemokine-modulating (CKM) regimen involving a TLR3 ligand (rintatolimod) and IFNα (which inhibits EP2 and EP4 expression and PGE2-driven CREB phosphorylation [106]), resulted in an intratumoral shift from Treg-attracting to CTL-attracting chemokines and local enhancements (up to 10-fold [148]) of the CTL markers in the TME of ovarian and breast cancer patients [148,149,150].

Additional research is needed to better understand the mechanism of regulation of inflammatory immune responses by BCG in resting and activated myeloid cells, the role of BCG strain-specific differences, histologic subtypes of bladder cancer, and the stage of the disease in immune and clinical responses to BCG. The identification of biomarkers predicting effective responses to BCG, analogous to those identified for PD1/PDL1 treatment [151,152,153], may help in the optimal selection of BCG dosage, duration, and most effective combinations for individual patients. Moreover, standardizing the biomarker assays poses another significant challenge. Different studies utilize various antibodies for PD-L1 staining, resulting in distinct staining patterns and intensities, which can lead to inconsistent results [61]. Factors such as tumor stage, grade, genetic mutations, and previous treatments can influence the immune response. Therefore, the predictive value of potential biomarkers must be validated across diverse patient cohorts to ensure their applicability to a broad range of patients. This includes stratifying patients based on relevant clinical and molecular characteristics to identify subgroups that may particularly benefit from biomarker-based predictive strategies. Addressing these challenges in properly designed prospective studies is critical to ensuring the reliability and widespread applicability to predict BCG responses and overcome resistance in bladder cancer patients.

## Figures and Tables

**Table 1:** PD-1/PD-L1 blockers trials in NMIBC.

Treatment	Study Phase	Trial	Reference
Nivolumab and BCG	Phase III	NCT04149574	[68]
Sasanlimab and BCG	Phase III	NCT04165317	[69]
Durvalumab and BCG or EBRT	Phase I	NCT03317158	[70]
Durvalumab and BCG	Phase III	NCT03528694	[71]
Durvalumab	Phase II	NCT03759496	[72]
Durvalumab and Tremelimumab	Phase I/II	NCT05120622	[73]
Durvalumab and S-488210/S-488211	Phase I/II	NCT04106115	[74]
Atezolizumab	Phase Ib/II	NCT02792192	[75]
